# Associations of dietary linoleic acid and alpha linolenic acid intake with cardiovascular, cancer and all-cause mortalities in patients with diabetes: NHANES 1999-2008

**DOI:** 10.3389/fcdhc.2024.1318578

**Published:** 2024-04-24

**Authors:** Mianmian Jiang, Huiping Zhu, Xiaoding Zhou, Xiaobing Zhai, Shiyang Li, Wenzhi Ma, Keyang Liu, Jinhong Cao, Ehab S. Eshak

**Affiliations:** ^1^ School of Public Health, Wuhan University, Wuhan, China; ^2^ School of Public Health, Capital Medicine University, Beijing, China; ^3^ Center for Artificial Intelligence Driven Drug Discovery, Faculty of Applied Sciences, Macao Polytechnic University, Macau, Macau SAR, China; ^4^ Public Health, Department of Social Medicine, Osaka University Graduate School of Medicine, Suita-shi, Japan; ^5^ Global Health Department, Denison University, Granville, OH, United States

**Keywords:** LA, ALA, NHANES, diabetes, CVD, cancer

## Abstract

**Objective:**

To investigate the association between the dietary intake of linoleic acid (LA) and alpha linolenic acid (ALA) with mortality outcomes in patients with diabetes.

**Participants:**

3,112 U.S. adults aged≥20 years.

**Setting:**

Basic information was collected at baseline of the National Health and Nutrition Examination Survey (NHANES). Serum CRP (mg/dL), total protein (g/L), waist circumference (cm), fasting blood glucose (mmol/L), white blood cell count, serum LDL-C, and serum HDL-C were also measured. Daily diets were also recorded using a 24-hour dietary review to produce the individuals’ intake of LA and ALA. The association between tertiles of LA and ALA intake with mortality was analyzed by weighted Cox models adjusted for the main confounders.

**Main outcome measures:**

The study included 3,112 adults with diabetes from the National Health and Nutrition Examination Survey (NHANES) from 1999 to 2008. Death outcomes were ascertained by linkage to the database records through 31 December 2015.

**Results:**

Subjects with a high intake of LA (T3) had 17% [hazard ratio (HR) 0.83, 95% CI 0.70 to 0.99) and 48% (HR=0.52, 0.35 to 0.80)] reductions in all-cause mortality and cardiovascular mortality, respectively, compared with subjects with lowest intake (T1). Similar results were observed for ALA, HR of cardiovascular mortality was 0.55 (0.38 to 0.81) and for all-cause mortality was 0.85 (0.69 to 1.04) comparing the highest to lowest intake tertiles.

**Conclusion:**

Higher intakes of LA and ALA were inversely associated with CVD and all-cause deaths in patients with diabetes. Proper dietary intakes of LA and ALA could contribute to the cardiovascular health and the long-term survival of patients with diabetes.

## Introduction

Type 2 diabetes mellitus (T2DM) accounts for 90%–95% of diabetes cases (1). The number of people with type 2 diabetes in the world is estimated to be 422 million and is expected to reach 642 million by 2040 (2). Worldwide, cardiovascular disease (CVD) affects approximately 32.2% of patients with T2DM (3). The prevalence of CVD among individuals with diabetes overall increased over the last 5 decades (4). In 2020, T2DM was the 9th leading cause of death globally according to the World Health Organization report (5).^5^ According to the American Heart Association (AHA), adults with T2DW have two to four times the risk of CVD morbidity and mortality as non-diabetic adults. In summary, diabetes is a major risk factor of CVD (3), which is also the most common cause of death in adults with T2DM (6).

Controlling blood glucose levels using dietary guidelines might play a role in modulating the pathogenesis of CVD in patients with T2DM. Dietary fat quality (i.e., the fatty acid composition) is a component of the diet that may affect the management of hyperglycemia in people with T2DM (7). For several decades, clinical trials and population-based studies have attempted to determine the effects of dietary intake of fatty acids on vascular outcomes and mortality; however, the results remain controversial and inconsistent. Linoleic acid (LA; 18:2n26) and alpha linolenic acid (ALA;18:3n23) belong to the n-6 (omega-6) and n-3 (omega-3) series of polyunsaturated fatty acids (PUFAs), respectively and are coming mainly from plant-based food sources (8) The current dietary guidelines for the prevention and management of CVD and the prevention of death among patients with T2DM recommend higher consumption of PUFAs at the expenses of the intake of saturated fatty acids, trans fats, and cholesterol (9). A large body of evidence supports a potential protective effect of marine PUFAs, on coronary heart disease (CHD) (10). However, fewer studies have evaluated how the plant-derived omega-3 PUFAs, such as ALA and omega-6 PUFA, such as LA relate to the risk of mortality from CVD and all-cause, and the results were inconsistent (11, 12), especially among patients with T2DM. Thus, this study aims to investigate the association between dietary intake of LA and ALA with CVD and other mortalities in patients with diabetes who have T2DM mostly.

## Methods

### Study population/data sources and study population

The National Health and Nutrition Examination Survey (NHANES) is a nationally representative survey of the noninstitutionalized civilian population in the United States. The details of sampling method and data collection have been published elsewhere (13).

The NHANES program began in the early 1960s and has been conducted as a series of surveys focusing on different population groups or health topics. In 1999, the survey became a continuous program that has a changing focus on a variety of health and nutrition measurements to meet emerging needs. We combined data from four waves of the NHANES, conducted between 1999 and 2008.

**Figure 1:**
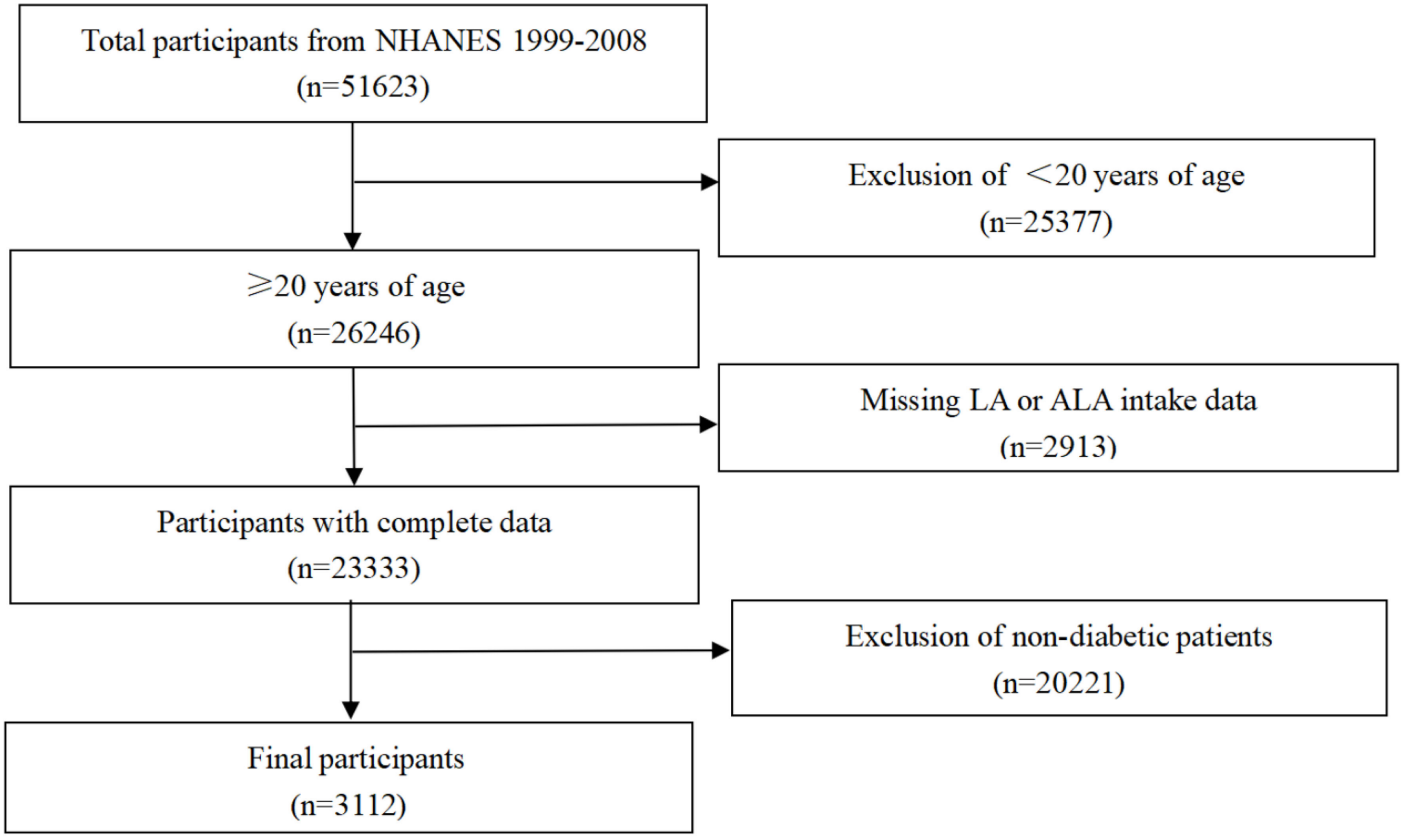
Flowchart of sample selection.

For our study, the population (total participants, n=51623) was limited to adults, ≥20 years of age (n=26246). After exclusion of cases with missing LA and ALA intake data (n=2913), only participants with diabetes were considered. Thus, data from 3,112 individuals were included in the final analysis (**Figure 1**). This longitudinal study was approved by the National Center for Health Statistics, and each participant provided a written consent.

### Ascertainment of diabetes, CVD, and covariates

Participants with fasting blood glucose≥7.8 mmol/L or glycated hemoglobin (HbA1c)≥6.5, and those being diagnosed by a physician to have diabetes or taking insulin or other diabetes medications are considered to be patients with diabetes.

Anthropometric data were collected by trained health technicians in the mobile examination center. The following covariates (part of the NHANES survey) were extracted for the analysis: serum CRP, total protein, fasting blood glucose, serum LDL-Cholesterol, serum HDL-Cholesterol, and white blood cell count and waist circumference as continuous variables. Age groups (<40, 40-60, and ≥60 years), gender (male or female), race (Non-Hispanic white, Non-Hispanic black, Mexican Americans, and others), educational level (<high school, high school, and >high school), BMI (<25, 25-30, and ≥30 kg/m^2^), alcohol consumption (never, moderate, and heavy drinkers), smoking (never, former, and current smokers), history of hypertension (yes or no), and history of hypercholesterolemia (yes or no) were used as categorical variables. For more information on obtaining these covariates, visit the NHANES website (https://www.cdc.gov/nchs/nhanes/).

Drinking status for each participant were grouped into never drinker (0 g/day), moderate drinker (0m/d<male<28gm/d, 0m/d<female<14gm/d), and heavy drinker (male≥28m/d, female≥14m/d). Smoking status was defined using two questions “smoking at least 100 cigarettes in a lifetime, and smoking now” and was divided into non-smokers (smoking less than 100 cigarettes in a lifetime), former smokers (smoking at least 100 cigarettes in a lifetime, but currently not smoking at all) and current smokers (smoking every day or sometimes). High blood pressure is defined as high blood pressure your doctor or other health professional tells you about. Hypercholesterolemia is defined as serum total cholesterol (mg/dL) ≥200 or if your doctor tells you have hypercholesterolemia. Nutrient intake was calculated by the United States Drug Administration’s Survey Nutrient Databases according to the 24-hour diet recall (14).

### Ascertainment of deaths

Mortality status was ascertained by probabilistic matching to the National Death Index through 31 December 2015 using a unique study identifier. More details of the matching method are available from the National Center for Health Statistics (15). We classified causes of deaths according to the codes of ICD-10 (international statistical classification of diseases, 10th revision). Primary outcomes for our study were mortality from CVD, cancer and all causes. We defined deaths from CVD as death from either heart diseases (codes I00-I09, I11, I13, I20-I51) or cerebrovascular diseases (codes I60-I69).

### Statistical analysis

The characteristics and distribution of the population were analyzed using descriptive statistics. Continuous variables are expressed as mean standard deviation and categorical variables as percentages. The Cox proportional hazard model was used to estimate the hazard ratio (HR) and 95% confidence interval (CI) to determine the association of LA and ALA intakes with all-cause mortality, CVD mortality, and cancer mortality. We evaluated the linear trend by entering the median of each group into the model. Model 1 was adjusted for age, and Model 2 was further adjusted for gender, race/ethnicity, education, BMI, and alcohol and tobacco use. Model 3 was further adjusted the total plasma protein (g/L), waist circumference (cm), fasting blood glucose (mmol/L), serum-low density lipoprotein cholesterol, serum-high density lipoprotein cholesterol, serum C-reactive protein (mg/dL), and white blood cell count. The hypertension history and hypercholesterolemia history were added in Model 4. Stratified analyses to determine whether the association of LA and ALA intakes with mortality outcomes were modified by age group (<60 years and ≥60 years), gender (males and females), smoking status (current smoker and non-current smoker), BMI (<25 years and ≥25kg/m^2^), history of hypertension (no or yes), and hypercholesterolemia (no or yes).

Since multiple comparisons can lead to the potential for type I error, the endpoints and subgroup analyses should be interpreted as exploratory analyses All analyses were performed using SAS version 9.4 and GraphPad Prism 8 software. Two-sided p values <0.05 were considered statistically significant.

## Results

### Population characteristics

**Table 1** shows the basic demographic characteristics of different groups of LA and ALA intake of participants in this study. Compared with the low intake group (T1) of LA, people in the higher groups (T2 and T3) were more likely to be male, non-Hispanic black, educated to high school or above, obese (BMI≥30.0), moderate and heavy alcohol users, current smokers, with larger waist circumference, and were more likely to have a history of high blood pressure. They were less likely to have a high serum HDL-C or protein. Among the three groups of ALA, compared with the low intake group (T1), the higher groups (T2 and T3) were more likely to be non-Hispanic black or Mexican American. There was no statistical difference in serum HDL-C content among the three groups, and the distribution of other basic conditions was basically the same as that of LA.

**Table 1:** Cohort characteristics at baseline for study participants according to tertiles of LA and ALA intake.

Characteristic	LA			*P* value	ALA			*P* value
	T1 <8.10	T2 8.10-14.63	T3 ≥14.63		T1 <0.78	T2 0.78-1.40	T3 ≥1.40	
Total (N)	1037	1037	1038		1029	1044	1039	
Age, *n*(%)				<0.001				<0.001
<40 y	54(8.1)	62(8.5)	105(12.7)		68(10.3)	61(7.9)	92(11.6)	
40-60 y	237(32.3)	302(38.7)	375(47.8)		259(35.2)	299(38.0)	356(46.5)	
≥60 y	746(59.6)	673(52.7)	558(39.5)		702(54.6)	684(54.2)	591(42.0)	
Gender, *n*(%)				<0.001				<0.001
Men	422(38.3)	504(48.7)	633(58.2)		421(39.1)	524(50.1)	614(56.6)	
Women	615(61.7)	533(51.3)	405(41.8)		608(60.9)	520(49.9)	425(43.4)	
Race/ethnicity, *n*(%)				0.008				0.003
Non-Hispanic white	225(8.0)	256(8.5)	249(7.8)		257(8.3)	261(8.4)	242(7.7)	
Non-Hispanic black	374(58.2)	382(60.9)	439(67.1)		355(56.0)	404(64.0)	436(66.4)	
Mexican American	283(17.9)	303(18.9)	265(14.7)		294(19.3)	296(17.9)	261(14.4)	
Other	125(15.9)	96(11.7)	85(10.4)		123(16.5)	83(9.7)	100(11.6)	
Education, *n*(%)				<.0001				<.0001
<High school	316(19.0)	258(13.8)	190(10.0)		310(19.0)	248(12.6)	206(10.4)	
High school	466(46.7)	449(45.5)	440(43.1)		458(46.3)	451(44.7)	446(44.1)	
>High school	255(34.4)	330(40.7)	408(47.3)		261(34.7)	345(42.7)	387(45.6)	
BMI, *n*(%)				<0.001				<0.001
Normal (<25.0 kg/m^2^)	223(20.9)	173(15.1)	164(15.3)		230(21.7)	152(14.8)	178(14.9)	
Overweight (25.0-30.0 kg/m^2^)	318(28.5)	326(28.0)	294(25.9)		305(27.6)	333(29.0)	300(25.7)	
Obese (≥30.0 kg/m^2^)	496(50.7)	538(56.9)	580(58.8)		494(50.8)	559(56.2)	561(59.4)	
Alcohol, *n*(%)				<0.001				<0.001
Never drinker	599(90.6)	575(84.1)	567(82.1)		592(90.2)	572(84.7)	577(80.8)	
Moderate drinking	33(5.2)	39(6.0)	71(9.2)		27(4.3)	51(7.4)	65(8.8)	
Heavy drinking	33(4.2)	56(10.0)	63(8.6)		35(5.5)	56(7.9)	62(9.4)	
Smoking, *n*(%)				0.033				0.067
Never smoker	523(48.9)	491(46.9)	461(48.5)		523(50.5)	485(44.8)	467(49.0)	
Former smoker	359(33.3)	356(33.2)	377(33.4)		347(31.3)	372(34.8)	373(33.6)	
Current smoker	154(17.9)	190(19.9)	197(18.1)		158(18.2)	187(20.5)	196(17.3)	
Serum CRP(mg/dL)^1^	0.7±1.6	0.6±1.0	0.6±1.1	0.143	0.7±1.2	0.7±1.5	0.6±1.0	0.330
Total protein (g/L)^1^	73.3±5.1	72.8±5.4	72.7±5.0	0.009	73.3±5.1	72.9±5.3	72.6±5.0	0.012
Waist circumference(cm) ^1^	104.6±15.3	106.2±15.3	108.5±16.5	<0.001	104.2±15.3	107.1±15.6	107.9±16.2	<0.001
Fasting Glucose (mmol/L) ^1^	6.7±2.6	6.7±2.6	6.9±2.8	0.114	6.8±2.7	6.7±2.4	6.9±2.9	0.162
Serum LDL-C(mg/dL) ^1^	109.4±22.8	109.3±23.2	109.9±24.5	0.828	109.4±23.3	109.5±23.1	109.8±24.1	0.929
White blood cell count^1^	7.5±2.1	7.6±2.6	7.6±2.3	0.621	7.5±2.1	7.6±2.6	7.7±2.3	0.132
Serum HDL-C(mg/dL) ^1^	48.5±14.1	48.4±13.5	47.0±13.3	0.022	48.7±14.0	47.7±13.3	47.6±13.6	0.116
History of hypertension, *n*(%)	693(64.6)	684(65.0)	603(57.0)	<0.001	684(65.5)	685(63.0)	611(57.9)	<0.001
History of hypercholesterolemia,*n*(%)	304(29.7)	302(29.1)	299(31.4)	0.967	301(29.9)	305(30.2)	299(30.2)	0.966

LA, linoleic acid; ALA, alpha linolenic acid; BMI, body mass index; LDL-C, low-density lipoprotein cholesterol; HDL-C, high-density lipoprotein cholesterol; CRP, C-reactive protein^1^.

Values are weighted mean ± SE for continuous variables or weighted % for categorical variables.

### Association between intake of LA and ALA and mortality

Among the 3112 participants who consumed LA, 1139 died during an median follow-up of 9.3 years (28,979 person-years), of which 240 died of CHD, 290 died of total CVD and 182 died of cancer.

**Table 2** shows the risks of all-cause mortality and CVD mortality were lower with higher intakes of LA and ALA. However, there was no association with cancer mortality. The multivariable HRs (95% CIs) for mortality from, CHD, total CVD, and all-cause in T3 group compared to T1 group of LA intake were 0.61 (0.40 to 0.94, *P*-trend=0.028), 0.52 (0.35 to 0.80, *P*-trend=0.003), and 0.83 (0.70 to 0.99, *P*-trend=0.036), respectively. Similar results were observed for ALA intake, where the multivariable HRs (95%CIs) for CHD, CVD, and all-cause mortality were 0.62 (0.40 to 0.97, *P*-trend=0.49) and 0.55 (0.38 to 0.81, *P*-trend=0.003), and 0.85 (0.69 to 1.04; *P*-trend= 0.087) respectively. Neither LA or ALA intake was associate with the risk of cancer mortality.

**Table 2:** Associations of LA and alpha ALA dietary intakes with cardiovascular, cancer, and all-cause mortalities in U.S. adults aged at least 20.

	LA			*P-*trend	ALA			*P-*trend
	T1 <8.10	T2 8.10-14.63	T3 ≥14.63		T1 <0.78	T2 0.78-1.40	T3 ≥1.40	
CHD mortality								
Deaths, *n*(%)	100(9.9)	83(7.1)	57(5.1)	0.002	100(9.6)	75(6.9)	65(5.5)	0.010
Deaths/person-years	587/9294	540/9561	403/10124		601/9283	495/9716	435/9979	
Unadjusted	1.00 [Reference]	0.71(0.51,0.98)	0.48(0.31,0.74)	0.002	1.00 [Reference]	0.74(0.52,1.05)	0.53(0.35,0.81)	0.005
Model 1	1.00 [Reference]	0.77(0.57,1.02)	0.58(0.39,0.87)	0.012	1.00 [Reference]	0.72(0.51,1.02)	0.61(0.40,0.92)	0.032
Model 2	1.00 [Reference]	0.79(0.57,1.09)	0.60(0.39,0.91)	0.020	1.00 [Reference]	0.73(0.50,1.07)	0.61(0.40,0.94)	0.040
Model3	1.00 [Reference]	0.77(0.55,1.08)	0.58(0.38,0.88)	0.013	1.00 [Reference]	0.72(0.49,1.05)	0.59(0.39,0.90)	0.024
Model 4	1.00 [Reference]	0.77(0.55,1.09)	0.61(0.40,0.94)	0.028	1.00 [Reference]	0.76(0.51,1.11)	0.62(0.40,0.97)	0.049
CVD mortality								
Deaths, *n*(%)	123(12.3)	103(8.7)	64(5.6)	<0.001	124(11.7)	95(8.7)	71(6)	<0.001
Deaths/person-years	729/9294	674/9561	439/10124		750/9283	625/9716	468/9979	
Unadjusted	1.00 [Reference]	0.70(0.53,0.92)	0.42(0.28,0.62)	<0.001	1.00 [Reference]	0.76(0.56,1.04)	0.48(0.34,0.69)	<0.001
Model 1	1.00 [Reference]	0.76(0.60,0.97)	0.51(0.35,0.75)	0.001	1.00 [Reference]	0.74(0.55,1.01)	0.55(0.39,0.79)	0.002
Model 2	1.00 [Reference]	0.78(0.60,1.02)	0.52(0.35,0.78)	0.002	1.00 [Reference]	0.75(0.54,1.05)	0.55(0.38,0.80)	0.003
Model3	1.00 [Reference]	0.77(0.58,1.01)	0.51(0.34,0.76)	0.001	1.00 [Reference]	0.74(0.53,1.03)	0.53(0.37,0.76)	0.001
Model 4	1.00 [Reference]	0.76(0.57,1.01)	0.52(0.35,0.80)	0.003	1.00 [Reference]	0.78(0.56,1.08)	0.55(0.38,0.81)	0.003
Cancer mortality								
Deaths, *n*(%)	61(4.5)	66(5.6)	55(4.6)	0.585	64(4.9)	61(6)	57(4)	0.777
Deaths/person-years	328/9294	417/9561	302/10124		349/9283	367/9716	330/9979	
Unadjusted	1.00 [Reference]	1.23(0.77,1.97)	0.95(0.59,1.53)	0.636	1.00 [Reference]	1.24(0.77,20)	0.79(0.49,1.26)	0.164
Model 1	1.00 [Reference]	1.31(0.83,2.07)	1.12(0.71,1.77)	0.803	1.00 [Reference]	1.20(0.75,1.93)	0.86(0.54,1.38)	0.371
Model 2	1.00 [Reference]	1.20(0.76,1.91)	1.05(0.67,1.65)	0.989	1.00 [Reference]	1.07(0.65,1.75)	0.79(0.48,1.30)	0.254
Model3	1.00 [Reference]	1.18(0.74,1.88)	1.03(0.65,1.62)	0.936	1.00 [Reference]	1.05(0.64,1.73)	0.77(0.47,1.26)	0.212
Model 4	1.00 [Reference]	1.18(0.73,1.91)	1.04(0.65,1.67)	0.995	1.00 [Reference]	1.08(0.66,1.78)	0.79(0.48,1.30)	0.253
All-cause mortality								
Deaths, *n*(%)	432(37.9)	403(33.5)	304(27.1)	<0.001	421(36.2)	387(34.1)	331(27.8)	<0.001
Deaths/person-years	2632/9294	2582/9561	1969/10124		6706/9283	7230/9716	7861/9979	
Unadjusted	1.00 [Reference]	0.88(0.72,1.06)	0.66(0.55,0.79)	<0.001	1.00 [Reference]	0.96(0.79,1.16)	0.72(0.59,0.88)	<0.001
Model 1	1.00 [Reference]	0.94(0.81,1.11)	0.80(0.67,0.94)	0.006	1.00 [Reference]	0.93(0.77,1.12)	0.81(0.66,0.99)	0.034
Model 2	1.00 [Reference]	0.99(0.84,1.16)	0.83(0.70,0.99)	0.030	1.00 [Reference]	0.97(0.80,1.17)	0.84(0.68,1.04)	0.090
Model3	1.00 [Reference]	0.99(0.84,1.16)	0.82(0.69,0.98)	0.021	1.00 [Reference]	0.96(0.79,1.16)	0.83(0.67,1.02)	0.065
Model 4	1.00 [Reference]	0.98(0.83,1.15)	0.83(0.70,0.99)	0.036	1.00 [Reference]	0.97(0.80,1.18)	0.85(0.69,1.04)	0.087

LA, linoleic acid. ALA, alpha linolenic acid.

Mode l: adjusted for age.

Model 2: Mode l +gender, race/ethnicity, education, body mass index, alcohol, and smoking.

Model 3: Model 2 + total protein, waist circumference, fasting blood glucose, serum low density lipoprotein cholesterol, serum high density lipoprotein cholesterol, serum C-reactive protein, and white blood cell count.

Model 4: Model 3 + history of hypertension and hypercholesterolemia.

### Stratification analysis between LA, ALA and all-cause, CVD and cancer mortalities in patients with diabetes

**Figure 2:**
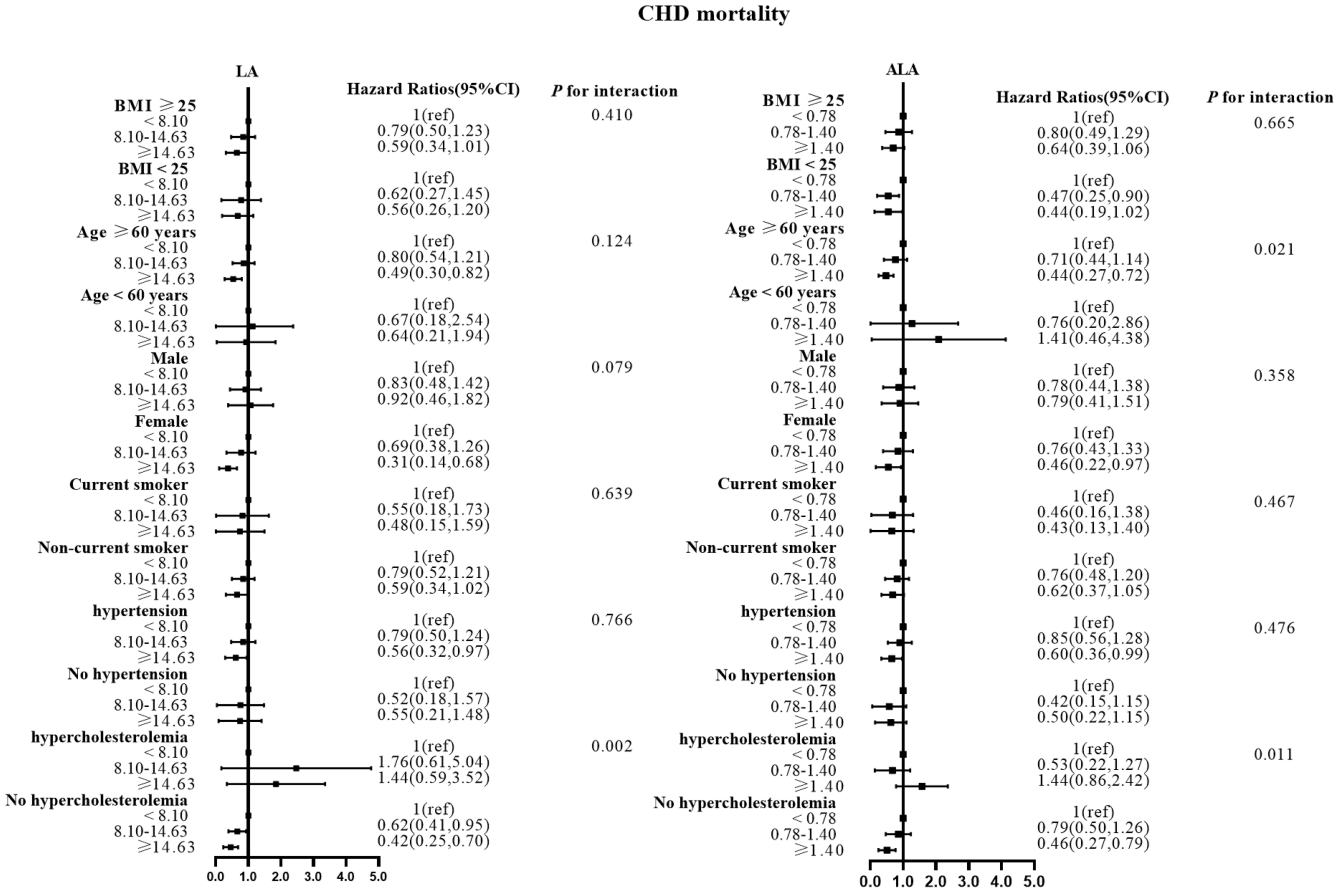
Associations between LA and ALA with CHD mortality in subgroups. Hazard ratios were adjusted for age, sex, race/ethnicity, education, BMI, alcohol, smoking, Total protein, Waist circumference(cm), Fasting Glucose, Serum LDL-cholesterol, Serum HDL-cholesterol, Serum C-reactive protein and White blood cell count, history of hypertension and history of hypercholesterolemia. CHD, coronary disease; LA, linoleic acid; ALA, alpha linolenic acid.

**Figure 3:**
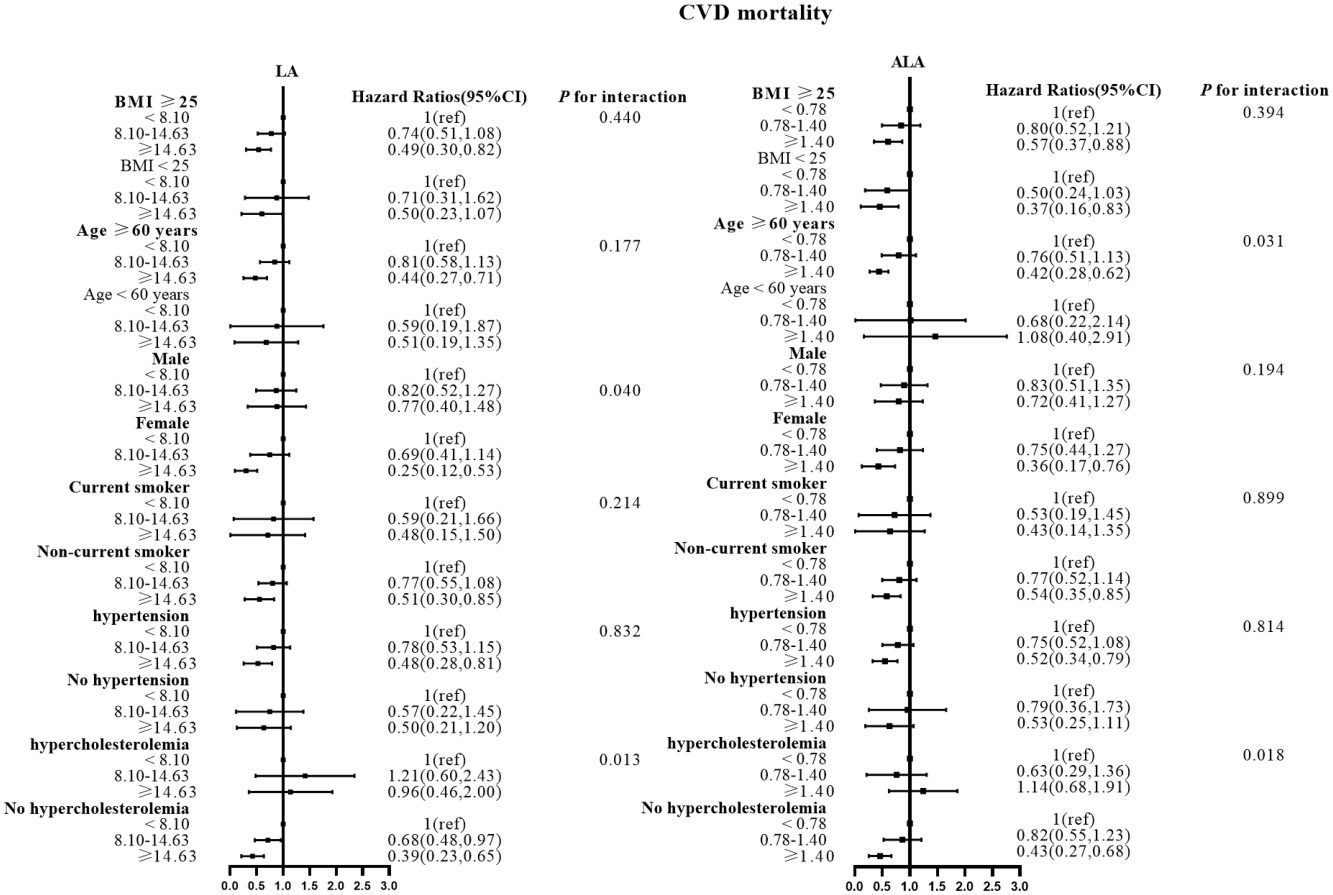
Associations between LA and ALA with CVD mortality in subgroups. Hazard ratios were adjusted for age, sex, race/ethnicity, education, BMI, alcohol, smoking, Total protein, Waist circumference, Fasting Glucose, Serum LDL-cholesterol, Serum HDL-cholesterol, Serum C-reactive protein and White blood cell count, history of hypertension and history of hypercholesterolemia. CVD, cardiovascular disease; LA, linoleic acid; ALA, alpha linolenic acid.

**Figure 4:**
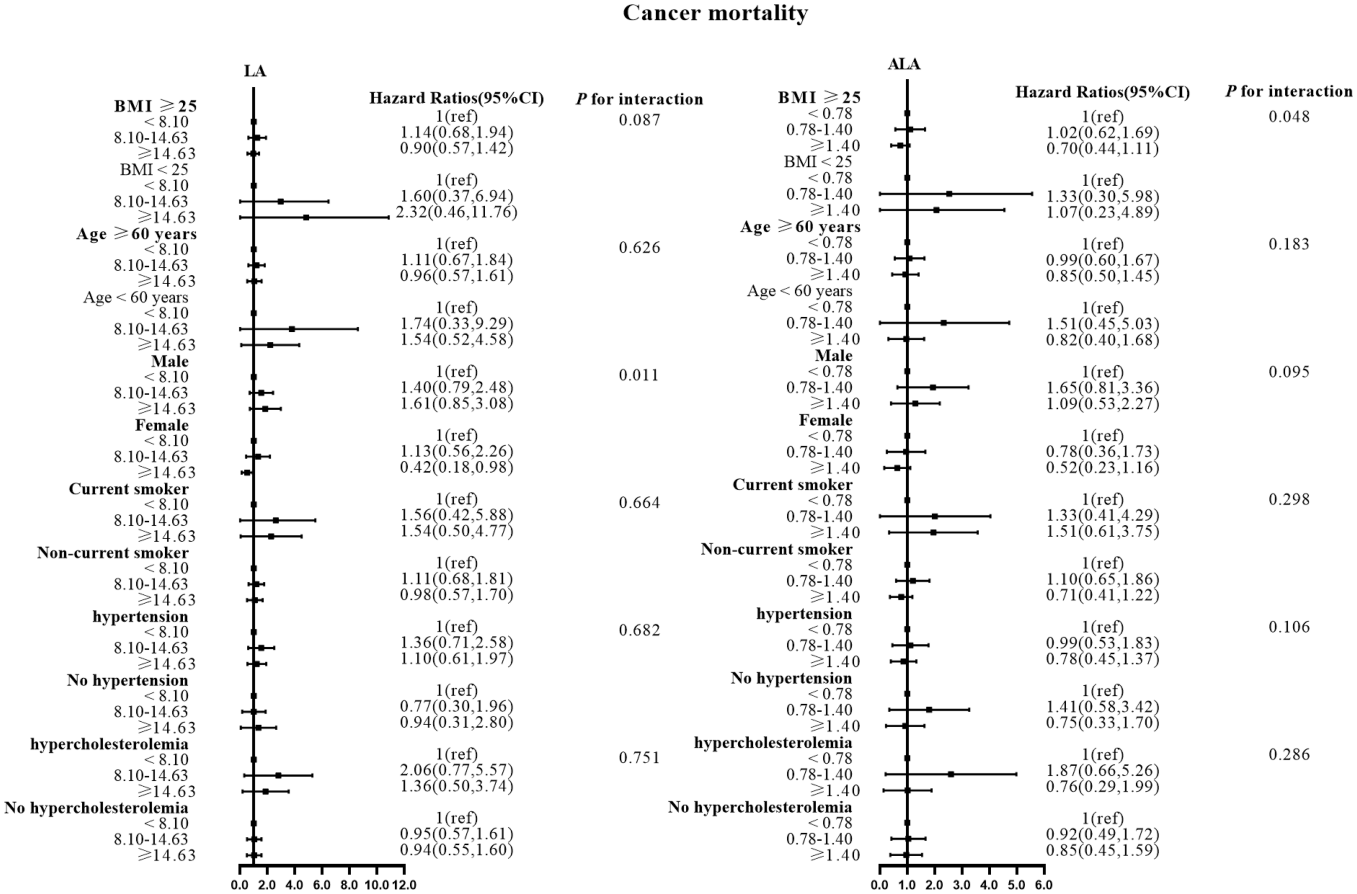
Associations between LA and ALA with Cancer mortality in subgroups. Hazard ratios were adjusted for age, sex, race/ethnicity, education, BMI, alcohol, smoking, Total protein, Waist circumference, Fasting Glucose, Serum LDL-cholesterol, Serum HDL-cholesterol, Serum C-reactive protein and White blood cell count, history of hypertension and history of hypercholesterolemia. LA, linoleic acid; ALA, alpha linolenic acid.

**Figure 5:**
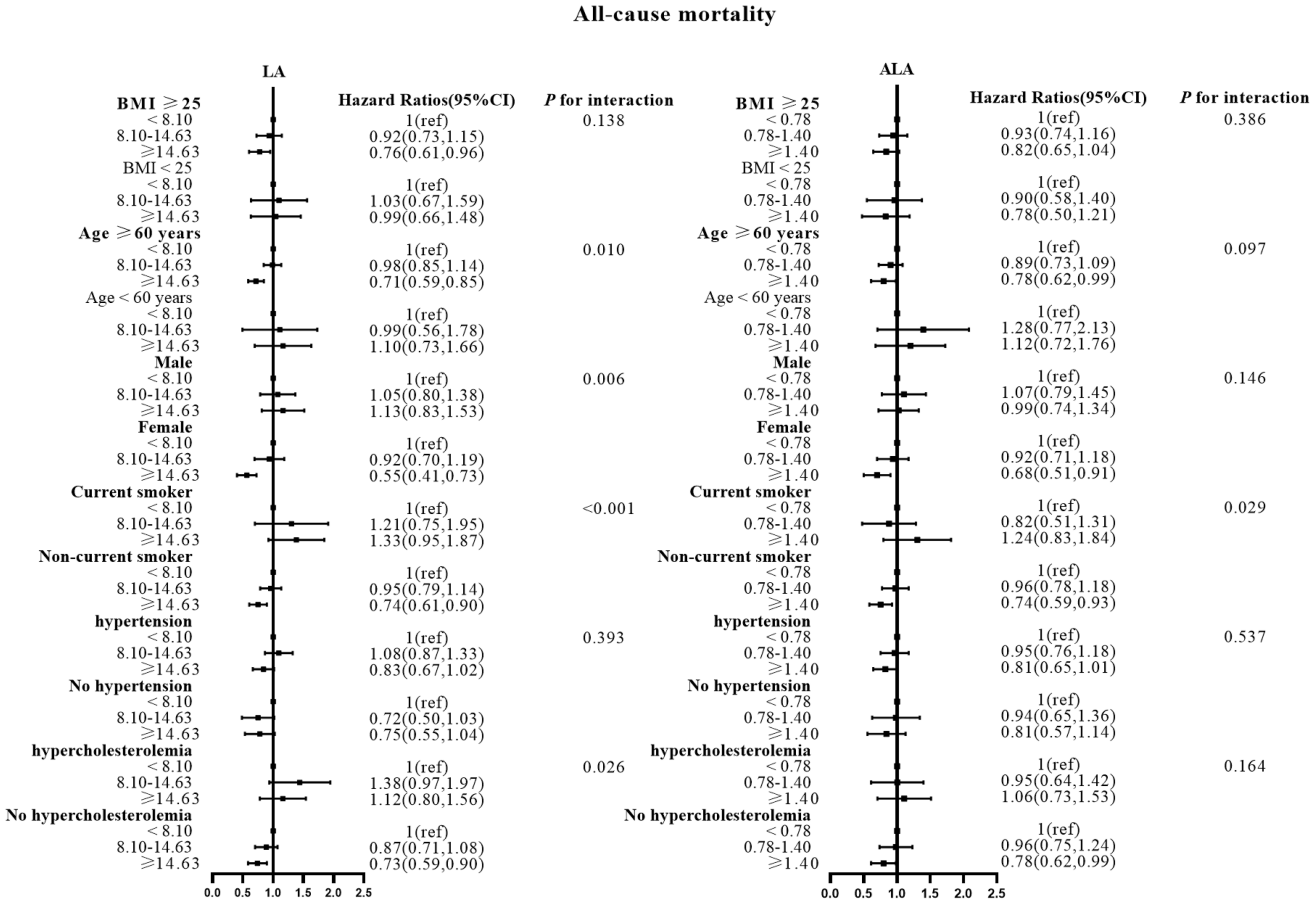
Associations between LA and ALA with All-cause mortality in subgroups. Hazard ratios were adjusted for age, sex, race/ethnicity, education, BMI, alcohol, smoking, Total protein, Waist circumference, Fasting Glucose, Serum LDL-cholesterol, Serum HDL-cholesterol, Serum C-reactive protein and White blood cell count, history of hypertension and history of hypercholesterolemia. LA, linoleic acid; ALA, alpha linolenic acid.

There were no effect modifications by the BMI or the history of hypertension for the associations of LA and alpha ALA intakes with mortality risks (*P*-interactions for all the mortality outcomes were >0.05). On the other hand, significant effect modifications were observed for age, sex, smoking status, and hypercholesterolemia status (**Figures 2**–**5**). The significant interactions for LA associations were observed mainly for risk of all-cause mortality (**Figure 5**) where the inverse association was evident for older (≥60 years), female, non-current smokers, and non-hypercholesterolemia participants. Whereas, the significant interactions for ALA associations were observed mainly for risk of CVD (**Figure 3**), including CHD mortality (**Figure 2**) where the inverse association was evident for younger (<60 years) and non-hypercholesterolemia participants. However, a significant interaction of LA associations, with a significant reverse association between female participants, was observed primarily for cancer mortality risk (**Figure 4**).

Also, the LA inverse associations with CVD and CHD mortalities were seen for women and non-hypercholesterolemia subjects, while the inverse association between ALA intake and all-cause mortality risk was seen in non-current smokers.

## Discussion

In this nationally representative cohort of the United States, the intake of LA and ALA was inversely associated with CHD, total CVD, and all-cause mortalities in diabetic participants, but were not significantly associated with cancer mortality. Age, sex, smoking status, and hypercholesterolemia status modified the observed associations, while the BMI and history of hypertension did not.

### Comparison with other studies and possible explanations

Although the associations between dietary intakes of LA and ALA with CVD mortality have been studied extensively in many studies; however, most studies were conducted in participants without chronic conditions, and the results are currently controversial.

Dietary intakes of LA were inversely associated with CVD mortality (7), but other studies have shown that there is no correlation (16). While, among Sydney Dietary Heart Study there was an increased risk of mortality with higher intakes of LA (17). Similarly, null (18), and positive (19) associations were observed between dietary intakes of ALA and risk of (all-cause or CVD) mortality. However, all these studies were conducted among the general populations, not among patients with diabetes.

Many studies have shown that dietary intake of n-3 polyunsaturated fatty acids is very beneficial for the health of patients with diabetes. Researchers have suggested that omega-3 polyunsaturated fatty acids from oily fish (long-chain omega-3 (LCn3), including eicosapentaenoic acid (EPA) and docosahexaenoic acid (DHA), as well as from plants (alpha-linolenic acid (ALA)) benefit cardiovascular health (20). Handelsman et al. over a period of follow-up found that n-3 polyunsaturated fatty acids significantly reduced all-cause mortality by 9% and all-cause mortality or CVD hospitalization by 8% for the primary endpoint (19). Similarly, baseline EPA, which is rich in n-3 polyunsaturated fatty acids, was found to be negatively correlated with a variety of cardiovascular risk biomarkers and all-cause mortality in patients with chronic heart failure in Sperling’s analysis of the study (21). Their protective effect on the heart is also very significant, the Japanese eat more fish than north Americans, the incidence of acute myocardial infarction, atherosclerosis and other ischemic diseases is low (22). But there is little evidence for much benefit from n-6 polyunsaturated fatty acids (23). Live experts are more focused on Omega-6/Omega-3 Fatty Acid Ratio and balance (16).

There are many investigations on diet of patients with diabetes. The present findings suggest that the Mediterranean (24), DASH, Portfolio, Nordic, and the vegetarian dietary patterns, may have positive effects on the risk of various cardiovascular disease outcomes (25). The DASH diet focuses on vegetables, fruits, whole grains, legumes, fat-free or low-fat dairy, and nuts and limits the intake of cholesterol, total and saturated fat, red and processed meats, sweets and added sugars, including sugar-sweetened beverages. Randomized controlled trials have shown that the DASH diet decreases LDL cholesterol, blood pressure, and other cardiometabolic risk factors. Prospective cohort studies have shown decreased diabetes and cardiovascular mortality in response to the DASH die (26). Similarly, Med Diet has a beneficial effect in reducing morbidity and mortality from a variety of cardiovascular diseases in the general population, including diabetic patients (24).

Several mechanisms explain the favorable association between LA, ALA, and CVD risk. Clinical trials of CVD outcomes in diabetic patients have reported that supplementation of n-3 fatty acids reduces triglyceride concentrations (27), improves arterial blood flow, and attenuates inflammatory signals (28). Another important function of n-3 PUFAs is the scavenging of free radicals, which diminishes inflammatory response and oxidation of lipoprotein particles, notably low-density lipoproteins. The interplay of these molecular processes has distinct cardioprotective effects, which involve actions on lipid metabolism, lipoprotein particle size, blood pressure, vascular function, coagulation potential, inflammatory response, atheroma formation, and antiarrhythmic (29). PUFAs were explained for making heart cells less impulsive by alteration of ion channels and to inhibit atrioventricular conduction and considerably slow down the possibility of having a prolonged QT interval (30). The alteration of potassium, sodium, and calcium channels demonstrating the antiarrhythmic effects (31), leading to the reduction of thromboxane synthesis, and the useful influences in context to heart rate variability. In conclusion, the protective effect of LA and ALA on cardiovascular disease is a complex and comprehensive process.

The inverse associations between dietary intakes of LA and ALA with mortality risks were more evident among older, female, non-smokers, and non-hypercholesterolemia subjects. The risk of diabetes associated with metabolic risk factors decreased significantly with age (32), with which the protective effect of LA and ALA intakes was not evident as it was for the older patients. The gender difference might be due to the different physiological metabolism patterns of LA and ALA in men and women. One of the mechanisms by which ALA can protect against CVD is its transformation inside the human body into ultra-long chain N–3 polyunsaturated fatty acids eicosapentaenoic acid (EPA) and Docosahexaenoic acid (DHA) which have potent protective effects on CVD (10). The endogenous conversion rate of ALA to EPA in women is generally higher than that in men (33). Smoking and hyperlipidemia are strong well-known risk factors for CVD and all-cause mortalities (34, 35). It is obvious that the protective effects of LA and ALA dietary intakes were insufficient to alleviate the smoking- or the hypercholesterolemia-induced increased risk of CVD and all-cause mortalities among diabetic patients.

### Strengths and limitations of study

The main advantages of this study include the use of nationally representative sample data, long-term follow-up visits, and examination of the relationship between LA and ALA and cardiovascular mortality in patients with diabetes, although there have not been many studies examining their association. In addition, the analysis included adjustment for multiple confounders and several subgroup analyses.

In this cohort study, NHANES performed a 24-hour retrospective nutritional survey, a convenient but limited method. However, this method was retrospective, and self-reported estimates of LA and ALA consumption were limited by measurement errors due to participant recall bias and incomplete nutrition database information. Second, NHANES1999-2008 relevant mortality profiles determine the cause of death by association with a national death index based on a death certificate. Although this approach has been validated by the Centers for Disease Control and Prevention (CDC) and is used in many CDC reports or related published reports, we cannot rule out the possibility of incorrect categorization of causes of death. Finally, although we have adjusted for confounders, there may be residual confounding.

## Conclusions

Our study found that the dietary intakes of LA and ALA were associated with reduced risk of CVD and all-cause mortalities in subjects with diabetes. While, we recommended higher intakes of LA and ALA acid from daily diet, the overall balance of fatty acids should be paid more attention to. Future studies need to reveal the mechanism of the correlation between LA and ALA intakes with the survival outcomes in diabetic participants.

## Data availability statement

Publicly available datasets were analyzed in this study. This data can be found here: https://www.cdc.gov/nchs/nhanes/.

## Author contributions

JC: Formal analysis, Supervision, Writing – review & editing. MJ: Data curation, Formal analysis, Investigation, Methodology, Writing – review & editing. XDZ: Formal analysis, Writing – original draft, Writing – review & editing. HZ: Formal analysis, Investigation, Methodology, Writing – original draft. XBZ: Data curation, Methodology, Writing – review & editing. SL: Data curation, Methodology, Writing – review & editing. WM: Data curation, Methodology, Writing – review & editing. KL: Data curation, Methodology, Writing – review & editing. EE: Data curation, Formal analysis, Methodology, Writing – review & editing.
